# Understanding Daily Care Experience Preferences Across the Lifespan of Older Adults: Application of Natural Language Processing

**DOI:** 10.1177/01939459241306946

**Published:** 2024-12-21

**Authors:** Se Hee Min, Kyungmi Woo, Jiyoun Song, Gregory L. Alexander, Terrence O’Malley, Maria D. Moen, Maxim Topaz

**Affiliations:** 1 University of Pennsylvania School of Nursing, Philadelphia, PA, USA; 2 Seoul National University College of Nursing, Seoul, South Korea; 3 Columbia University School of Nursing, New York, NY, USA; 4 Harvard University, Cambridge, MA, USA; 5 ADVault Inc., Richardson, TX, USA

**Keywords:** aging, care preference, older adults, patient-centeredness

## Abstract

**Introduction:**

Older adults are a heterogeneous group, and their care experience preferences are likely to be diverse and individualized. Thus, the aim of this study was to identify categories of older adults’ care experience preferences and to examine similarities and differences across different age groups.

**Methods:**

The initial categories of older adults’ care experience preferences were identified through a qualitative review of narrative text (n = 3134) in the ADVault data set. A natural language processing (NLP) algorithm was used to automatically and accurately define older adults’ care experience preference categories. Descriptive statistics were used to examine similarities and differences in care experience preference categories across different age groups.

**Results:**

The overall average performance of NLP algorithms was relatively high (average *F*-score = 0.88; range: 0.77-0.96). Through a qualitative review of 350 randomly selected texts, a total of 11 categories were identified. The most frequent category was music, followed by photographs, entertainment, family/friends, religion-related, atmosphere, flower/plants, pet, bed/bedding, hobby, and other. After applying the best performing NLP algorithm to each category, older adults’ care experience preference categories were music (41.32%), followed by photographs (28.47%), entertainment (13.46%), religion-related (n = 12.69%), pet (12.22%), flower/plants (11.51%), family/friends (8.45%), atmosphere (7.75%), bed/bedding (6.12%), and hobby (5.45%). Young-old and old-old adults had similar care experience preferences with music being the most frequent category while old-old adults had photographs as the most frequent category for their care experience preference.

**Conclusion:**

Clinicians must understand the distinct categories of care experience preferences and incorporate them into personalized care planning.

The number of older adults aged 65 years and over is expected to double to 2.1 billion by 2050 worldwide.^1^ Many older adults are living longer with disabling medical conditions and require significant assistance to manage their daily health.^2^ Chronic disease and disabilities remain as one of the significant challenges for older adults, with an estimated 80% living with 1 or more chronic diseases and 20% with disability.^3^ Person-centered care has been emphasized to ensure high-quality care for older adults.^4^ Herein, person-centered care is defined as “health care that reflects patient’s likes, needs, and preferences which leads to informed decision making.”^5^ However, a considerable gap between the care provided and the care experience of older adults exists, which impedes the delivery of patient-centered care.^6^ Several factors contribute to this issue, including a shortage of resources for assessing and delivering patient-centered care, restricted professional autonomy for clinicians, and the often prioritized medical complexity of older adults.^6-8^ Consequently, clinicians pay little attention to understanding older adults’ care preferences, which are often dismissed and lack the attributes of person-centeredness.^6-8^ This oversight may lead to dissatisfaction with care and poor health outcomes such as impaired health-related quality of life.^7-9^ This highlights the importance of bridging the gap between the care offered and the preferences of older adults, which can be achieved by first gaining a comprehensive understanding of their diverse care preferences.^10^

Care experience is a measure of patient-centeredness and includes various interactions and experiences that patients have with the health care system.^11^ This encompasses the quality of care received, the setting in which the care is delivered, and the relationships formed with clinicians.^11,12^ Positive care experience has been associated with a higher engagement with adherence to care and health outcomes.^11^ Thus, it is important to understand the different care experience preferences of older adults, which are likely to be unique based on individuals’ health and functional status.^13^ These care experience preferences reflect how older adults prefer to have their care needs met and help to identify favorable things that bring them happiness in daily care experience.^13^ When care experience preferences are fulfilled, older adults experience a sense of subjective well-being and happiness, creating motivation to engage in life.^13,14^ The tenet of person-centered care is understanding older adults’ care experience preferences regardless of their care setting (ie, community, hospital, long-term care setting), yet these preferences are often overlooked for various reasons unique to each environment.^5,15^ For instance, older adults residing in the community may face greater challenges in communicating their care experience preferences related to their health care visits and/or home health care services than those in long-term care settings.^16^ This is due to the limited opportunities to express their needs and preferences, as they are not in continuous care environments. Consequently, their care experiences are frequently overlooked, making it more problematic for them to receive the personalized attention they deserve. Another challenge is that older adults are often perceived as a relatively homogeneous group (eg, physically unwell, multiple comorbidities), which remains a significant barrier to person-centered care.^17^ Rather, older adults must be considered a heterogeneous group.^13^ Accordingly, care experience preferences are also likely to be diverse and individualized among older adults. Thus, it is vital to gather reliable data on older adults’ care experience preferences and incorporate this information into individualized care plans.^13^ Valuing these care experience preferences empowers older adults to be more involved in their care decisions, enhancing their sense of autonomy and dignity.^13,14^ Furthermore, this knowledge can help clinicians engage in meaningful discussions about care experience preferences, aligning them with actual care arrangements.^18^

Older adults can be classified into 3 life stage subgroups: young-old (age 65-74 years), old-old (age 75-84 years), and oldest-old (age 85 years and over).^19^ Previous studies often examined older adults as a whole to understand their medical care or end-of-life preferences.^20^ Yet, significant differences were found in the sociodemographic and clinical characteristics of these 3 groups such as increased institutionalization and comorbidities, all of which could affect their daily likes, needs, and preferences.^21^ For example, young-old adults are more likely to suffer from anxiety and depression than old-old adults and might prefer to listen to music for coping.^22^ In contrast, the oldest-old adults suffer from more severe functional disability and may prefer active family involvement in care.^23^ Regardless of life-stage, older adults may prefer to have photographs of friends and family that will promote social connectedness and bring them joy and happiness into care.^24^ Thus, it is essential to understand the similarities and differences in care experience preferences of older adults across different life-stages. This knowledge will serve as a foundation for person-centered care and further help older adults achieve the highest level of physical, mental, and psychosocial well-being.^4^

To date, a commonly used tool to assess care experience preferences is the Preferences for Everyday Living Inventory (PELI) completed by clinicians.^25^ While PELI is a comprehensive and validated tool,^25^ it is lengthy and often not completed due to diminished cognitive capacity of older adults or time constraints of the clinicians.^26^ Thus, using PELI might not fully reflect older adults’ care experience preferences with the issue of incompleteness or limited administration,^26^ plus it could increase the documentation burden that clinicians experience.^26,27^ To address these research gaps, the current study uses the ADVault data, an interactive online person-centered platform, that allows older adults to create, access, and share their plan of care which ensures that their health care preferences are clearly communicated with their clinicians.^28^ Additionally, older adults can articulate their care preferences through narrative text, offering richer, more self-reflective insights than the standardized answers typically gathered from survey questionnaires.^29^ These narrative texts have been considered as reliable source to explore patient-centered care concepts, such as patient care experience preferences,^29^ with the use of natural language processing (NLP) to improve the accuracy of study findings.^30^ Furthermore, since the platform allows older adults to express their care preferences in their own words at any time,^28^ the problem of not evaluating these preferences due to limited clinician availability is significantly reduced.

The purpose of this study was to identify categories of older adults’ care experience preferences through a qualitative review of narrative text contained in ADVault. Our goal is to develop and test a NLP algorithm to automatically and accurately define care experience preference categories and to examine similarities and differences in older adults’ care experience preference choices across different age groups.

## Methods

### Design and Data Set

This is a secondary data analysis of data from ADVault, Inc (dba MyDirectives, Inc, Richardson, TX, USA), a digital advance care planning platform that is populated by individuals who want to express their goals, preferences, and priorities for care in advance of a health crisis or emergency that would render them unable to communicate directly with the care team. Individuals can access ADVault through clinician referral, direct registration, invitations by health care organizations, and during a health care visit and are able to complete the surveys independently or with assistance from their caregivers if needed. The ADVault data set was de-identified for use by the study and maintains a standardized, interoperable structure for purposes of data exchange and accessibility. The main goal of ADVault in this study and for this project, coupled with the mission of the Moving Forward Coalition, is to better enable patient-centered care through understanding older adults’ care preferences, and later further expanding the project to assist the Moving Forward Coalition to develop a foundational tool to gather care preferences of older adults residing in nursing homes. This interactive online person-centered ADVault platform allows easy access to advance care planning information and easily facilitates communication of these care experience preferences between clinicians and patients across various care settings.

### Study Sample

A random sample of text representing care preferences from 10 000 community-dwelling adults across the United States was obtained between January 2020 and December 2022. This represented a self-selected population that wanted their care experience and treatment preferences understood by their families and clinicians, and who were able to make their preferences known. We removed younger adults (age 64 years and below) and those who did not share their care preferences. After applying these exclusion criteria, 3134 statements about older adults’ care experience preferences were used for data analysis.

### Measures

#### Sample characteristics

Responses from a standardized questionnaire were used to analyze the sample’s sociodemographic characteristics such as age, gender, and care preferences such as importance at end of life, what clinicians should know about the individual’s religion, faith, spirituality, willingness for palliative care consult, terminal illness treatment, incapacitation treatment, and preferences for final days.

#### Care experience preferences

Community-dwelling older adults answered the following question in free text: “Describe the things that bring you joy. Photographs or other items you would like to have nearby, or music you’d like to hear. A favorite pillow, a night light, or your favorite flowers.” Responses to this question were assessed by our team using NLP methods.

### Ethical Considerations

The ADVault data set, codebook, and survey questionnaires were completely de-identified, downloaded to a secure, encrypted server at the Columbia University School of Nursing. The de-identified information was archived and analyzed for the current study. The current study received the Columbia University Institutional Review Board’s declaration of exemption.

### Data Analysis

#### Development of initial categories of care experience preferences

The research team initially identified categories of older adults’ care experience preferences through a qualitative review of 350 randomly selected texts representing <10% of the entire the ADVault data set (n = 3134). This methodology involved several stages of inductive thematic analysis, including becoming familiar with the data, generating initial codes, identifying preliminary categories, reviewing and refining categories, and defining the final categories documented in an Excel spreadsheet.^31^ First, the 2 experts (PhD-prepared nurses) reviewed the current literature on older adults’ care preferences from various health research databases (ie, PubMed, Embase, Web of Science, CINAHL, PsycINFO) to understand important categories to be considered in the following steps.^31^ Second, they reviewed the first 150 texts among the randomly selected 350 texts to develop a preliminary list of categories based on the content shared by older adults.^31^ These experts then gathered and discussed the preliminary list of categories until a consensus was reached.^31^ A third expert was involved in helping achieve a consensus when there was a discrepancy between the 2 experts.^31^ This process was repeated for 3 rounds (first 150 texts, second 100 texts, and third 100 texts) until a consensus was achieved on the final categories of older adults’ care preferences.^31^

#### NLP algorithm creation and testing

Utilizing NLP facilitates extraction of meaningful insights from narrative texts by identifying categories, themes, trends, sentiment, and patterns within the data and uncovering hidden relationships that might be overlooked by conventional statistical approaches.^32^ In addition, another benefit of NLP is the significant reduction in human bias by automating text processing, which leads to more accurate and reliable study findings.^33^ Thus, as part of our methodology, we utilized a machine learning approach to develop an NLP algorithm, which is a computer-based system capable of extracting meaning from textual information.^32^ This approach uses statistical and computational techniques to analyze and understand human language, which allows the algorithm to process, interpret, and generate language-based data.^32^ Our study employed the NLP algorithm to identify and categorize specific concepts within text data. By training the algorithm to recognize patterns, associations, and semantic relationships in the text, the algorithm could accurately identify and classify instances of the identified categories.^34^

We used the previously annotated data set to test whether an NLP algorithm can identify categories of older adults’ preferences as accurately as a clinical expert. As previously described, 2 experts manually assigned labels to this preliminary data set (n = 350). Assigning “yes” indicated the presence of the concept of identified categories within the text data. As a first step of developing the NLP algorithm, preprocessing was conducted to ensure the text data’s quality and consistency before further analysis.^35^ The process began by cleaning the text and removing extraneous characters such as punctuation marks, special symbols, and numeric digits, which are irrelevant for semantic analysis.^35^ We then tokenized the text data, which divides a text into smaller units, such as individual words and tokens, to perform later analysis.^35^ To reduce noise in the data and to focus the analysis on meaningful words, we removed stopwords, commonly occurring words in the language that have little semantic meaning, such as “the,” “is,” and “and,” from the data.^35^ Then, we standardized and normalized the text data by converting all words to lowercase to ensure consistency and to prevent the algorithm from treating different words with different cases as distinct entities.^35^ As a final step, we transformed the preprocessed text data into numerical vectors using techniques such as Term Frequency-Inverse Document Frequency, enabling further evaluation and algorithm development within the NLP workflow.^36^

Following the splitting of the data set into training (70%) and testing (30%) subsets, which enabled the development of the NLP algorithm using a substantial portion of the data, while also setting aside a separate validation set for unbiased evaluation, we proceeded to apply traditional machine learning algorithms for the classification task (ie, whether the categories were accurately captured within text or not).^32^ Specifically, logistic regression, support vector machine, random forest, and gradient-boosted tree algorithms were utilized on the training set to discern patterns and relationships within the text data.^32^ These 4 distinct algorithms were selected because each 1 offers its own set of strengths and weaknesses in addressing specific challenges in language, including ambiguity, context, and variability, and thus will allow us to identify the best performing algorithm to fulfill our study aim.^32^ For example, logistic regression is a linear classification algorithm suitable for binary classification tasks while support vector machine can also handle nonlinear relationships and complex NLP tasks.^37^ The performance of these algorithms on the preliminary data set was evaluated to determine each algorithm’s effectiveness in accurately categorizing text according to each identified care preference. The best performing algorithm with the highest performance—measured through performance metrices such as precision (eg, the positive predictive value defined as the number of true positives out of the total number of predicted positives), recall (eg, sensitivity defined as the number of true positives out of an actual number of positives), and *F*-score (eg, the weighted harmonic mean of the precision and recall) was selected.^38^ All analyses were implemented using the Konstanz Information Miner (KNIME) software, version 2.1 (KNIME, Konstanz, Germany).

#### Identification of care experience categories within the entire data set and across age groups

The finalized best performing NLP algorithm, which varied by categories, was applied to the entire ADVault data set (N = 3134). Descriptive statistics were used to calculate the frequency of older adults’ care experience preferences categories across different age groups: young-old adults (age 65-74 years), old-old adults (age 75-84 years), and oldest-old adults (age 85 years and over). In addition, participant characteristics were obtained and compared using analysis of variance or chi-squared tests where *P*-values less than .05 were considered statistically significant differences. All analyses were implemented using the R software, version 4.1.0 (R Foundation of Statistical Computing, Vienna, Austria).

## Results

### Participant Characteristics

Our sample of 3134 participants comprised 2107 young-old adults (67.2%), 890 old-old adults (28.4%), and 137 oldest-old adults (4.4%). The mean age of young-old adults was 69.86 years (standard deviation [SD] 2.53), old-old adults 78.19 years (SD 2.63), and oldest-old adults 88.78 years (SD 4.01) (*P* < .0001). More than half of the adults were female for young-old adults (62.94%), old-old adults (55.17%), and oldest-old adults (59.40%) (*P* = .002). More than 75% of the sample across all age groups valued quality of life priorities similarly, emphasizing the importance of being pain-free, surrounded by family, and retaining self-care abilities in feeding, bathing, and overall personal care. Additionally, they highlighted the significance of not imposing financial or physical burdens on their families and avoiding prolonged reliance on life-support machines or artificial feeding through tubes. Young-old adults (96.06%), old-old adults (96.85%), and oldest-old adults (97.81%) were willing to consider palliative care consultations (*P* = .37). In addition, the majority of young-old adults (79.30%), old-old adults (82.47%), and oldest-old adults (83.94%) preferred clinicians to stop all life-sustaining treatments for their terminal illness treatment (*P* = .35). More than half of young-old adults (59.16%), old-old adults (56.17%), and oldest-old adults (58.39%) wanted to spend their final days at home (*P* = .053). Table 1 details sample characteristics.

**Table 1.** Sample Characteristics.

Characteristics	Young-old (age 65-74 years)	Old-old (age 75-84 years)	Oldest-old (age ≥ 85 years)	All	*P*-value
Sample size, n (%)	2107 (67.20)	890 (28.40)	137 (4.40)	3134 (100)	
Age, mean (SD), years	69.86 (2.53)	78.19 (2.63)	88.78 (4.01)	73.05 (5.67)	< .001*
Gender, n (%)					.002*
Male	781 (37.06)	399 (44.83)	57 (41.60)	1237 (39.47)	
Female	1324 (62.94)	491 (55.17)	80 (59.40)	1895 (60.53)	
Importance, n (%)					.153
Being free from pain	1802 (85.52)	772 (86.74)	118 (86.13)	2692 (85.89)	
Being with my family	1563 (74.18)	685 (76.96)	105 (76.64)	2353 (75.07)	
Being able to feed, bathe, and take care of myself	1570 (74.51)	680 (76.40)	100 (72.99)	2350 (74.98)	
Not being a financial burden to my family	1707 (81.01)	718 (80.67)	105 (76.64)	2530 (80.72)	
Not being a physical burden to my family	1681 (79.78)	720 (80.89)	107 (78.10)	2508 (80.02)	
Being at peace with my God	1242 (58.94)	546 (61.34)	88 (64.23)	1876 (59.85)	
Resolving conflicts	647 (30.70)	291 (32.69)	43 (31.38)	981 (31.30)	
Avoiding prolonged dependence on machines	1835 (87.09)	798 (89.66)	123 (89.78)	2756 (87.93)	
Avoiding prolonged dependence on artificial or assisted nutrition through tubes	1763 (83.67)	756 (85.28)	117 (85.40)	2636 (84.10)	
Dying at home	849 (40.29)	362 (40.67)	66 (48.17)	1277 (40.74)	
Caregivers to know about religion, faith, spirituality, n (%), (yes)	1342 (63.69)	555 (62.35)	89 (64.96)	1986 (63.36)	.863
Willingness for palliative care consult, n (%), (yes)	2024 (96.06)	862 (96.85)	134 (97.81)	3020 (96.36)	.372
Terminal illness treatment, n (%)					.353
I prefer that they stop all life-sustaining treatments	1671 (79.30)	734 (82.47)	115 (83.94)	2520 (80.40)	
I would like them to keep trying life-sustaining treatments for selected period	23 (1.09)	9 (1.01)	1 (0.72)	33 (1.05)	
I would like them to keep trying life-sustaining treatments indefinitely	33 (1.56)	8 (0.89)	0 (0.00)	41 (1.30)	
I would like them to keep trying life-sustaining treatments based on Healthcare Advocates (HCA)	184 (8.73)	67 (7.52)	13 (9.48)	264 (8.42)	
Other/Refused	196 (9.32)	72 (8.11)	8 (5.86)	276 (8.83)	
Incapacitation treatment, n (%)					.117
I prefer that they stop all life-sustaining treatments	1842 (87.42)	807 (90.67)	120 (87.59)	2769 (88.35)	
I would like them to keep trying life-sustaining treatments for selected period	15 (0.71)	5 (0.56)	0 (0)	20 (0.63)	
I would like them to keep trying life-sustaining treatments indefinitely	31 (1.47)	8 (0.89)	0 (0)	39 (1.24)	
I would like them to keep trying life-sustaining treatments based on HCA	82 (3.89)	22 (2.47)	9 (6.56)	112 (3.57)	
Other/Refused	137 (6.51)	48 (5.41)	8 (5.85)	193 (6.21)	
Final days, n (%)					.053
At home	1256 (59.61)	500 (56.17)	80 (58.39)	1836 (58.58)	
In the hospital	87 (4.12)	25 (2.80)	8 (5.83)	120 (3.82)	
In a hospice facility	361 (17.13)	182 (20.44)	20 (14.59)	563 (17.96)	
Not sure	338 (16.04)	163 (18.31)	26 (18.97)	527 (16.81)	
Refused	65 (3.1)	20 (2.28)	3 (2.22)	88 (2.83)	

*P-value <.05.

Abbreviation: SD: standard deviation.

### Initial Categories of Care Experience Preferences

Through a qualitative review of 350 randomly selected texts, a total of 11 categories were identified. The most frequent category was music, including classical, rock, and calm music. The next most frequent category was photographs, followed by entertainment, family/friends, religion-related, atmosphere, flower/plants, pet, bed/bedding, hobby, and other. Table 2 details the initial categories with examples.

**Table 2.** Initial Categories of Care Preferences.

Frequency rank	Category name	Examples
1	Music (n = 178)	Classical, rock, calm music
2	Photographs (n = 94)	Photographs of friends, family
3	Entertainment (passive) (n = 84)	Watching movies, listening to radios
4	Family/friends (n = 74)	Spouse, grandchildren, son, daughter
5	Religion-related (n = 66)	Bible, prayer, church, gospel
6	Atmosphere (n = 59)	Nature, fan, candle, oil
7	Flower/plants (n = 57)	Lilies, roses, daisies
8	Pet (n = 55)	Cat, dog
9	Bed/bedding (n = 38)	Pillow, blanket, bed facing window
10	Hobby (active) (n = 37)	Gardening, crocheting, traveling
11	Other (n = 26)	Warmth, holding hands, massage

### NLP-Applied Categories of Care Experience Preferences

We applied the best performing NLP algorithm, which varied by categories, to the entire ADVault data set (N = 3134). Herein, we excluded the “other” category with small frequency (less than 10% of total) in the previous step, which may lead to issues such as overfitting or random effects especially when validation is limited due to small sample size (Rajput et al, 2023).^39^ The overall average performance of NLP algorithms was relatively high (average *F*-score = 0.88; range: 0.77-0.96). After applying the best performing NLP algorithm to each category, the most frequent category remained the same as the initial category: music (41.32%), followed by photographs (28.47%), entertainment (13.46%), religion-related (n = 12.69%), pet (12.22%), flower/plants (11.51%), family/friends (8.45%), atmosphere (7.75%), bed/bedding (6.12%), and hobby (5.45%). Table 3 details the NLP categories of care preferences applied to the entire data set. Supplemental Table 1 details the performance metrics for each NLP algorithm.

**Table 3.** NLP-Applied Categories of Care Preferences.

Frequency rank	Category name	Best performing algorithm	Unlabeled texts (%) (n = 3134)	New NLP-applied frequency rank
1	Music (n = 178)	Random forest	n = 1295 (41.32%)	1
2	Photographs (n = 94)	Support vector machine/random forest	n = 892 (28.47%)	2
3	Entertainment (passive) (n = 84)	Support vector machine	n = 422 (13.46%)	3
4	Family/friends (n = 74)	Support vector machine	n = 265 (8.45%)	7
5	Religion-related (n = 66)	Gradient boost	n = 398 (12.69%)	4
6	Atmosphere (n = 59)	Support vector machine	n = 243 (7.75%)	8
7	Flower/plants (n = 57)	Gradient boost	n = 361 (11.51%)	6
8	Pet (n = 55)	Random forest	n = 383 (12.22%)	5
9	Bed/bedding (n = 38)	Gradient boost	n = 192 (6.12%)	9
10	Hobby (active) (n = 37)	Gradient boost	n = 171 (5.45%)	10

We excluded the “other” category with small frequency (less than 10% of total) in the previous step, which may lead to issues such as overfitting or random effects especially when validation is limited due to small sample size.

Abbreviation: NLP: natural language processing.

### Age Group–specific Care Experience Preferences

Young-old and old-old adults had similar care experience preferences, while old-old adults had different care preferences in terms of frequency. Young-old adults identified music (49.35%) as the most frequent care experience preference category, followed by photographs (31.37%), entertainment (15.51%), religion-related (14.61%), flower/plants (14.52%), pet (14.00%), atmosphere (9.77%), bed/bedding (7.64%), family/friends (6.92%), and hobby (3.84%). Similarly, old-old adults identified music (47.86%), photographs (31.12%), entertainment (16.62%), flower/plants (13.03%), religion-related (12.80%), pet (9.77%), atmosphere (8.08%), bed/bedding (7.64%), family/friends (4.83%), and hobby (3.03%), whereas, for oldest-old adults, the most frequent care experience preference category was photographs (34.30%), followed by music (32.11%), flower/plants (16.78%), entertainment (15.32%), religion-related (14.59%), atmosphere (10.20%), pet (9.48%), bed/bedding (8.02%), family/friends (5.10%), and hobby (3.64%). Table 4 details the care experience preference categories for each age group.

**Table 4.** Age Group–specific Care Preferences.

Frequency rank	Category name (n = 3134)	Young-old (%) (n = 2107)	New NLP-applied frequency rank	Old-old (%) (n = 890)	New NLP-applied frequency rank	Oldest-old (%) (n = 137)	New NLP-applied frequency rank
1	Music	n = 1040 (49.35%)	1	n = 426 (47.86%)	1	n = 44 (32.11%)	2
2	Photographs	n = 661 (31.37%)	2	n = 277 (31.12%)	2	n = 47 (34.30%)	1
3	Entertainment (passive)	n = 327 (15.51%)	3	n = 148 (16.62%)	3	n = 21 (15.32%)	4
4	Religion-related	n = 308 (14.61%)	4	n = 114 (12.80%)	5	n = 20 (14.59%)	5
5	Pet	n = 295 (14.00%)	6	n = 87 (9.77%)	6	n = 13 (9.48%)	7
6	Flower/plants	n = 306 (14.52%)	5	n = 116 (13.03%)	4	n = 23 (16.78%)	3
7	Family/friends	n = 146 (6.92%)	9	n = 43 (4.83%)	9	n = 7 (5.10%)	9
8	Atmosphere	n = 206 (9.77%)	7	n = 72 (8.08%)	7	n = 14 (10.20%)	6
9	Bed/bedding	n = 161 (7.64%)	8	n = 68 (7.64%)	8	n = 11 (8.02%)	8
10	Hobby (active)	n = 81 (3.84%)	10	n = 27 (3.03%)	10	n = 5 (3.64%)	10

Abbreviation: NLP: natural language processing.

## Discussion

This is the first study to use NLP to identify older adults’ care experience preferences and examine the similarities and differences across different age groups. The identified categories were music, photographs, entertainment, religion-related, pet, flower/plants, family/friends, atmosphere, bed/bedding, and hobby in frequency. When examining these categories across different age groups, young-old and old-old adults had similar care experience preferences, while those of old-old adults differed.

Older adults placed high importance on music as a part of their care experience regardless of age. This music category mainly consisted of listening to classical, rock, and calm music. Our study aligns with previous research that identifies music as an important outlet, especially for older adults with limited mobility and functional ability to improve their mood.^40^ Consequently, music therapy, such as playing instruments or listening to different types of music, has been actively used as a safe and effective intervention in clinical settings for older adults with mood disorders and dementia.^41^ For example, a recent randomized controlled trial found a significant between-group difference in cognitive function and depression among older adults who received usual care plus receptive music therapy intervention over time.^42^ Overall, music enriches the lives of older adults by fostering emotional health, supporting cognitive abilities, and encouraging social engagement, which emphasizes the importance of routinely offering music into their daily care routine.^43^ For older adults receiving sporadic care in the community, clinicians might consider playing background music tailored to their preferences, ensuring it does not interfere with the care being provided.^44^ Furthermore, integrating music into care could be beneficial for older adults, especially in long-term care facilities such as nursing homes, where music can trigger memories and emotions that help nursing home residents connect with their past.^45,46^ This connection can enhance their mood and alleviate feelings of loneliness and depression.^45^ Such emotional engagement is particularly advantageous for nursing home residents, who are often prone to social isolation and declining mental health.^45^ Given the positive impact of music on older adults’ well-being and quality of life, it is important for clinicians to incorporate music into their care plan and offer daily music-related activities.

In addition, our study identified several categories of personal possessions, including photographs of friends and family, flowers/plants, and bed/bedding. Older adults regarded photographs as the most important category of their care experience preferences among all personal possessions. Personal possessions often reflect an individual’s personality and interest, contributing to the preservation of their identity as they age.^47^ In addition, photographs of friends and family can offer emotional support, which can serve as reminders of cherished memories.^47^ For older adults, personal possessions are more than mere physical items; they significantly enhance their emotional, psychological, and social well-being.^47^ Clinicians may engage family members or caregivers to accommodate community-dwelling older adults’ preferences regarding the placement of their personal belongings, such as keeping them next to their bed, especially for those who may be too frail or have mobility issues to manage this themselves.^48^ The importance of personal possessions extends to older adults in both hospital and long-term care settings, where personal belongings can establish a private space and provide older adults with the chance to relax.^49-51^ For example, similar to our study findings, nursing home residents valued photographs and paintings that often depicted children, grandchildren, deceased spouses, weddings, and other loved ones among all personal possessions brought to their rooms.^52^ However, the rules for bringing personal belongings upon admission to nursing homes often vary or nursing home residents are too ill to communicate their preferences upon admission, which has remained a significant barrier to delivering high-quality care.^52^ Thus, nursing home clinicians should ensure effective communication about permitted possessions during admission and consistently communicate with residents once their health has stabilized to give them ample opportunity to express their care experience preferences and accommodate them as much as possible, thus enabling delivery of personalized care. This issue is also relevant in hospital settings, where specific guidelines may exist regarding what items are permitted based on patients’ conditions.^50,51^ As most hospitalizations are unplanned and unexpected, it is essential for patients and their families to maintain ongoing communication with clinicians throughout their hospital stay. This communication should be informed based on the patient’s condition and can help clarify what personal possessions are recommended or prohibited for safety and hygiene purposes.^50,51^

When comparing age-group differences, young-old adults placed music, photographs, and entertainment as their top 3 categories for care experience preferences. In contrast, oldest-old adults showed more importance to their surroundings and identified atmosphere, such as candles, oil, and nature, more frequently than entertainment. Older adults tend to have limited contact with nature due to limited mobility and access in their care settings, which significantly differ from what they were accustomed to when they were more independent.^53^ With aging, older adults have become more accustomed to doing passive activities such as watching television, listening to the radio, and reading books, which have also been important tools for maintaining their quality of life.^54^ Yet, contact with nature has been shown to significantly improve overall health and health-related quality of life, physical strength, mood and cognition, and socialization among older adults.^55^ Older adults living in the community should be encouraged to begin with simple nature-based activities, such as listening to natural sounds, and gradually progress to more active pursuits, such as gardening and spending time outdoors, depending on their functional abilities.^55^ For older adults in long-term care settings, clinicians should consider assessing the need to develop simple, targeted, nature-based programs based on their resources to enhance person-centered care and older adults’ well-being. Incorporating such care preferences allows for a more person-centered approach to care delivery, promoting a greater sense of autonomy and dignity among older adults.^55^

While previous studies have highlighted the crucial role of family and friends in providing emotional support to older adults in everyday life,^56,57^ our study found family and friends to be ranked lower in terms of care experience preferences. This shift may be attributed to a decline in social activities and reduced engagement with family and friends as individuals age, even among those who were socially active and family-oriented in their younger years of old adulthood.^58^ Such changes often stem from changes in their social roles and emerging health issues that restrict their mobility, thereby impacting their ability to maintain close relationships with family and friends.^58^ However, it should also be noted that the framing of the main question included examples such as photographs, music, pillows, night lights, and flowers, which may have significantly shaped the responses of older adults. This question might have led participants to prioritize these possessions over interpersonal relationships such as family and friends.

Overall, our study findings could extend to serve as a foundational knowledge base to assist the Moving Forward Coalition in developing a comprehensive tool for gathering the care preferences of older adults living in nursing homes. This tool could empower nursing home residents by ensuring their voices are heard and their care experience preferences are met, ultimately enhancing the quality of care they receive and promoting a more person-centered approach in long-term care settings. In addition, by integrating these care experience preferences into care plans, the Moving Forward Coalition can foster an environment that respects the unique needs of older adults and lead to improved care satisfaction and well-being. Yet, there are several limitations to consider. First, the sample size for each age group differs significantly. While we did not conduct propensity score matching due to a significant loss of sample size in both young-old and old-old adults, future research with a larger sample size may consider using propensity score matching in terms of important covariates for matched analysis among all 3 groups.^59^ This approach will allow for more reliable and generalizable study findings for older adults across different age groups.^59^ Second, our sample consisted of older adults. Due to the lack of digital literacy in older adults, our sample could have missed those with low digital literacy, thus limiting the generalizability of our study findings. Third, our study was limited to older adults aged 65 and above. Preferences for care experiences are likely to vary due to several key factors, including differences in health and functionality among older adults.^21^ These aspects should be carefully considered in future research. Fourth, the main question included examples such as photographs, music, pillows, night lights, and flowers, which may have influenced the responses of older adults. Therefore, our findings should be interpreted with caution. Last, we identified categories of care experience preferences based on frequency using NLP. Future research should aim to consider identifying meaningful themes within the categories that will help build more personalized care plans.

## Conclusion

This study identified frequent categories of care experience preferences among older adults and similarities and differences in those same preferences across young-old, old-old, and oldest-old adults. Clinicians must understand the distinct categories of care preferences and incorporate them into personalized care planning. In addition, using these older adults’ developed quality indicators for daily care experience preference is essential in delivering high-quality care.

## Supplemental Material

sj-docx-1-wjn-10.1177_01939459241306946 – Supplemental material for Understanding Daily Care Experience Preferences Across the Lifespan of Older Adults: Application of Natural Language Processing Supplemental material, sj-docx-1-wjn-10.1177_01939459241306946 for Understanding Daily Care Experience Preferences Across the Lifespan of Older Adults: Application of Natural Language Processing by Se Hee Min, Kyungmi Woo, Jiyoun Song, Gregory L. Alexander, Terrence O’Malley, Maria D. Moen and Maxim Topaz in Western Journal of Nursing Research
