# Supplementary material for: Understanding Daily Care Experience Preferences Across the Lifespan of Older Adults: Application of Natural Language Processing
Source: West J Nurs Res. 2024 Dec 21;47(2):71–81. doi: 10.1177/01939459241306946 (PMC11742706; doi:10.1177/01939459241306946)
Supplement: sj-docx-1-wjn-10.1177_01939459241306946 – Supplemental material for Understanding Daily Care Experience Preferences Across the Lifespan of Older Adults: Application of Natural Language Processing [file sj-docx-1-wjn-10.1177_01939459241306946.docx]

**Supplementary Table 1.** NLP Algorithm Performance Metrices for Categories of Care Preferences

| **Machine learning method** | **Recall** | **Precision** | **Sensitivity** | **Specificity** | **F-score** | **Accuracy** | **AUC-ROC** |
| --- | --- | --- | --- | --- | --- | --- | --- |
| **Music** | | | | | | | |
| Support Vector Machine | 0.859 | 0.864 | 0.859 | 0.859 | 0.860 | 0.861 | 0.9218 |
| Random Forest | 0.880 | 0.882 | 0.880 | 0.880 | 0.889 | 0.881 | 0.9090 |
| Gradient Boost | 0.838 | 0.847 | 0.838 | 0.838 | 0.839 | 0.880 | 0.9206 |
| Logistic Regression | 0.849 | 0.856 | 0.849 | 0.849 | 0.850 | 0.851 | 0.9053 |
| **Photos** | | | | | | | |
| Support Vector Machine | 0.968 | 0.958 | 0.968 | 0.968 | 0.963 | 0.970 | 0.9726 |
| Random Forest | 0.968 | 0.958 | 0.968 | 0.968 | 0.963 | 0.970 | 0.9736 |
| Gradient Boost | 0.961 | 0.943 | 0.961 | 0.961 | 0.951 | 0.960 | 0.9797 |
| Logistic Regression | 0.768 | 0.924 | 0.768 | 0.768 | 0.808 | 0.871 | 0.9831 |
| **Entertainment** | | | | | | | |
| Support Vector Machine | 0.827 | 0.898 | 0.827 | 0.827 | 0.885 | 0.901 | 0.8858 |
| Random Forest | 0.813 | 0.917 | 0.813 | 0.813 | 0.850 | 0.901 | 0.9166 |
| Gradient Boost | 0.840 | 0.884 | 0.840 | 0.840 | 0.859 | 0.901 | 0.9129 |
| Logistic Regression | 0.540 | 0.884 | 0.540 | 0.540 | 0.508 | 0.772 | 0.8842 |
| **Family/friends** | | | | | | | |
| Support Vector Machine | 0.799 | 0.864 | 0.799 | 0.799 | 0.825 | 0.891 | 0.8898 |
| Random Forest | 0.743 | 0.896 | 0.743 | 0.743 | 0.788 | 0.881 | 0.8651 |
| Gradient Boost | 0.724 | 0.802 | 0.724 | 0.724 | 0.752 | 0.851 | 0.8924 |
| Logistic Regression | 0.545 | 0.899 | 0.545 | 0.545 | 0.527 | 0.802 | 0.8921 |
| **Religion-related** | | | | | | | |
| Support Vector Machine | 0.844 | 0.931 | 0.844 | 0.844 | 0.879 | 0.931 | 0.8963 |
| Random Forest | 0.850 | 0.965 | 0.850 | 0.850 | 0.894 | 0.941 | 0.9682 |
| Gradient Boost | 0.900 | 0.976 | 0.900 | 0.900 | 0.932 | 0.960 | 0.8802 |
| Logistic Regression | 0.525 | 0.905 | 0.525 | 0.525 | 0.495 | 0.812 | 0.8864 |
| **Atmosphere** | | | | | | | |
| Support Vector Machine | 0.764 | 0.793 | 0.764 | 0.764 | 0.777 | 0.881 | 0.8690 |
| Random Forest | 0.717 | 0.813 | 0.717 | 0.717 | 0.751 | 0.881 | 0.8676 |
| Gradient Boost | 0.729 | 0.753 | 0.729 | 0.729 | 0.740 | 0.861 | 0.8631 |
| Logistic Regression | 0.559 | 0.924 | 0.559 | 0.559 | 0.564 | 0.851 | 0.7976 |
| **Flower/plants** | | | | | | | |
| Support Vector Machine | 0.788 | 0.915 | 0.788 | 0.788 | 0.834 | 0.921 | 0.8354 |
| Random Forest | 0.782 | 0.877 | 0.782 | 0.782 | 0.819 | 0.911 | 0.9482 |
| Gradient Boost | 0.841 | 0.900 | 0.841 | 0.841 | 0.866 | 0.931 | 0.8936 |
| Logistic Regression | 0.500 | 0.821 | 0.500 | 0.500 | 0.711 | 0.832 | 0.9391 |
| **Pet** | | | | | | | |
| Support Vector Machine | 0.894 | 0.916 | 0.894 | 0.894 | 0.905 | 0.950 | 0.9787 |
| Random Forest | 0.957 | 0.935 | 0.957 | 0.957 | 0.945 | 0.970 | 0.9949 |
| Gradient Boost | 0.976 | 0.900 | 0.976 | 0.976 | 0.932 | 0.960 | 0.9971 |
| Logistic Regression | 0.500 | 0.801 | 0.500 | 0.500 | 0.703 | 0.842 | 0.9735 |
| **Bed/bedding** | | | | | | | |
| Support Vector Machine | 0.932 | 0.851 | 0.932 | 0.932 | 0.886 | 0.950 | 0.9677 |
| Random Forest | 0.903 | 0.939 | 0.903 | 0.903 | 0.920 | 0.970 | 0.9798 |
| Gradient Boost | 0.989 | 0.923 | 0.989 | 0.989 | 0.953 | 0.980 | 0.9939 |
| Logistic Regression | 0.435 | 0.511 | 0.435 | 0.435 | 0.528 | 0.891 | 0.9475 |
| **Hobby** | | | | | | | |
| Support Vector Machine | 0.671 | 0.796 | 0.671 | 0.671 | 0.711 | 0.911 | 0.6717 |
| Random Forest | 0.545 | 0.950 | 0.545 | 0.545 | 0.557 | 0.901 | 0.8424 |
| Gradient Boost | 0.756 | 0.806 | 0.756 | 0.756 | 0.778 | 0.921 | 0.8020 |
| Logistic Regression | 0.422 | 0.500 | 0.500 | 0.500 | 0.382 | 0.891 | 0.7808 |
